# Letter to the Editor: “Retinal oculomics and risk of incident aortic aneurysm and aortic adverse events: a population-based cohort study”

**DOI:** 10.1097/JS9.0000000000003015

**Published:** 2025-07-08

**Authors:** Ling Ling, Yuchen Mei, Xuanyu Yang, Yawen Hao

**Affiliations:** a Nanchang Bright Eye Hospital, Nanchang, Jiangxi Province, People’s Republic of China; b School of Ophthalmology and Optometry, Jiangxi Medical College, Nanchang University, Nanchang, Jiangxi, People’s Republic of China

*Dear Editor,*

We have read with great interest the article titled *“Retinal oculomics and risk of incident aortic aneurysm and aortic adverse events: a population-based cohort study”* published in the *International Journal of Surgery*^[1]^. This study introduces an innovative approach by employing retinal vascular features (RVFs) as biomarkers for early detection of aortic aneurysms (AA) and related adverse events. The application of a fully automated Retina-based Microvascular Health Assessment System (RMHAS) to quantify multidimensional RVFs, including branching angle, caliber, complexity, density, length, and tortuosity, demonstrates the potential of integrating artificial intelligence with ophthalmic imaging for systemic health assessment. In line with the TITAN 2025 guideline on AI transparency^[2]^, a generative AI-based language model was used solely for language polishing and had no bearing on the scientific content.

The prospective design of this large-scale cohort study, utilizing data from the UK Biobank with a median follow-up of 11.0 years, provides robust evidence supporting the association between specific RVFs and the incidence of AA and aortic adverse events. The findings that retinal arterial branching angle and venular caliber variability are significantly associated with incident AA, and that venular angular asymmetry correlates with aortic adverse events, offer promising insights into non-invasive screening methods for AA. Moreover, the study’s stratification of RVFs by AA subtypes adds depth to our understanding of the differential impact of RVFs on abdominal and thoracic aortic aneurysms.

However, we believe that certain aspects of the study could be further refined to enhance the clarity and applicability of the findings. Firstly, while the study adjusted for several potential confounders, including age, sex, body mass index, smoking status, diabetes, hypertension, and hyperlipidemia, the inclusion of additional variables such as cholesterol levels and genetic risk scores could provide a more comprehensive assessment of the factors influencing AA risk. For instance, previous research has demonstrated that each unit increase in body mass index correlates with a higher risk of cardiovascular diseases, including AA, with a hazard ratio of 1.10 (95% confidence interval: 1.05–1.15) per unit increase in BMI. Incorporating such continuous variables into the multivariable models could offer a more nuanced understanding of the relationships between RVFs and AA risk^[3,4]^.

Secondly, the study’s focus on RVFs as predictors of AA is commendable; however, the differential impact of RVFs on abdominal and thoracic aortic aneurysms warrants further exploration. The findings that venular caliber is associated with an increased risk of abdominal aortic aneurysm (hazard ratio: 1.21) and that arterial complexity, length, and tortuosity are related to decreased risk of thoracic aortic aneurysm (hazard ratios ranging from 0.77 to 0.82) suggest that the pathophysiological mechanisms underlying these two types of aneurysms may differ. Further research is needed to elucidate these differences and to determine whether RVFs can serve as subtype-specific biomarkers for AA^[5]^.

Additionally, the study’s reliance on data from the UK Biobank, which predominantly includes individuals of European descent, may limit the generalizability of the findings to other populations. Cross-cultural studies have shown significant variations in retinal vascular features among different ethnic groups, which could influence the applicability of RVFs as biomarkers for AA in non-European populations. Therefore, conducting multi-center, cross-national studies is essential to validate the findings and to assess the universal applicability of RVFs in AA risk prediction.

In conclusion, while the study provides valuable insights into the potential of RVFs as biomarkers for early detection of aortic aneurysms, further refinement of the analysis, including the consideration of additional risk factors, exploration of subtype-specific associations, and validation across diverse populations, is necessary to enhance the clinical applicability of these findings. We commend the authors for their innovative approach and look forward to future research that will build upon these promising results.

## Data Availability

No new data were generated or analyzed.

## AI assistance

A generative AI-based language model was employed solely for grammar and style editing.
